# To benefit tomorrow’s children: female participation in Brazilian eugenics, 1918-1936

**DOI:** 10.1590/S0104-59702024000100023

**Published:** 2024-05-17

**Authors:** Thayná Soares de Almeida Vieira

**Affiliations:** i Doutoranda, Programa de Pós-graduação em História das Ciências e da Saúde/Casa de Oswaldo Cruz/Fiocruz. Rio de Janeiro – RJ – Brasil thaynasoaresalmeida@gmail.com

**Keywords:** Eugenics, Gender, Medical discourse, Eunice Penna Kehl (1901-1980, Ítala Silva de Oliveira (1897-1984, Eugenia, Gênero, Discursos médicos, Eunice Penna Kehl (1901-1980, Ítala Silva de Oliveira (1897-1984

## Abstract

This text analyzes female participation in Brazilian eugenics and medical discourse on the roles and social functions of the sexes during the first half of the twentieth century. In examining the production of two women, Ítala Silva de Oliveira and Eunice Penna Kehl, we maintain that certain women were effectively engaged in the eugenics movement and worked to bring women closer to eugenics. This analysis makes it possible to explore a pedagogical dimension of eugenics and of the popularization of this movement by attempting to form a hygienist and eugenist consciousness among women.

## Resumo

O texto analisa a participação feminina na eugenia brasileira e os discursos médicos acerca dos papéis e funções sociais dos sexos na primeira metade do século XX. A partir da análise da produção de duas mulheres, Ítala Silva de Oliveira e Eunice Penna Kehl, o objetivo principal é argumentar que determinadas mulheres se engajaram efetivamente no movimento eugênico, trabalhando em prol da aproximação feminina com a eugenia. Tal análise permite explorar uma dimensão pedagógica e de popularização da eugenia por meio da tentativa de formação de uma consciência higiênica e eugênica nas mulheres.

A eugenia desempenhou papel fundamental nos projetos de modernização e nos processos de intervenção na vida social e na biologia humana no início do século XX (Souza, Wegner, 2018). Entendida como a ciência da hereditariedade humana, a eugenia associou-se estreitamente com as discussões acerca de temas como evolução, seleção natural e social, progresso e degeneração, prometendo criar "uma inovadora engenharia racional capaz de produzir um novo homem, eliminando as imperfeições hereditárias e estimulando o desenvolvimento de suas habilidades físicas e mentais" (p.328). Nesse contexto, a ciência eugênica apresentava um conjunto de medidas que reiteravam a crença de que os problemas sociais poderiam ser resolvidos com a intervenção da ciência.

No caso do Brasil, é importante ressaltar que a eugenia não foi inserida simplesmente como um reflexo do modelo praticado em outros países. Alinhando-se à ideia de que a eugenia é, ao mesmo tempo, uma ciência e um movimento social intrinsecamente nacionalista (Adams, 1990), é possível afirmar que ela adquiria características próprias em cada contexto nacional. Assim, no Brasil, a ciência eugênica inseriu-se como uma resposta à preocupação das elites políticas e intelectuais com as chamadas "questões sociais", como o mau estado de saúde da população, as condições sanitárias e a composição racial da nacionalidade (Souza, 2008).

Em razão do clima tropical e da população mestiça, o Brasil era a representação do que os europeus consideravam disgênico. Assim, os intelectuais brasileiros buscaram adaptar a ciência eugênica para o cenário do país e, segundo Stepan (2004), com isso, começaram a exibir uma espécie de "otimismo realista" em relação à população. Realista, pois não negavam que os brasileiros eram biologicamente inferiores, mas começaram a desafiar o que era considerado a causa da inferioridade.

O projeto de modernização do Brasil pensado pelos eugenistas, pautado principalmente no desejo de retirar o país de um atraso civilizacional, almejava a transformação do Brasil em um país de indivíduos aptos ao exercício pleno da cidadania, ou seja, um país fundamentalmente de trabalhadores, sendo essa a condição de saúde do novo cidadão (Cupello, 2013). Assim, durante a primeira metade do século XX, as mulheres, entendidas pelos eugenistas como as guardiãs das futuras gerações, tornaram-se objeto central das ações dos movimentos de higiene social, principalmente em decorrência das preocupações com as questões de hereditariedade e de formação dessa nação de cidadãos saudáveis e aptos ao trabalho.

Conforme apontado por Ruíz Somavilla e Jiménez Lucena (2003), existem simetrias e assimetrias quando se pensa em gênero que dificultam discernir se as diferenças entre homens e mulheres em questão de comportamento, de estilo cognitivo e de personalidade são genuínas ou fruto de expectativas sociais. Segundo as autoras, a ciência, supostamente precisa e objetiva, desempenhou papel de destaque em relação à desigualdade de gênero, legitimando estudos que trabalham pela manutenção de clichês de gênero e normatizando condutas a partir de uma perspectiva única. A eugenia foi uma dessas ciências que trabalhou em prol da manutenção dos papéis de gênero socialmente impostos por meio de um discurso científico supostamente neutro, objetivo e racional

No discurso eugênico, a mulher e a família eram alvos centrais de preocupação por parte do Estado brasileiro, sendo possível perceber uma insistência nos benefícios da maternidade.

Conforme apontado por Foucault (1980), a moral e a ética defendidas pela eugenia se estabeleciam pela noção médica da higiene, que definia a moralidade baseada nas noções do homem-pai e da mulher-esposa-mãe integrantes da instituição do casamento. Nesse contexto, a família e o matrimônio configuravam-se como instituição higiênica e espaço eugênico capazes de promover uma educação baseada em princípios morais saudáveis para as próximas gerações. Como mãe e geradora da criança que viria a ser o "futuro da nação", as mulheres eram então uma parte de grande importância para o projeto de modernização proposto pelos médicos para a sociedade, a fim de que se pudessem garantir proles sadias e o suposto aprimoramento racial.

Assim, as questões de raça e a defesa de um suposto futuro eugênico da nação foram a base para elaboração de discursos e práticas que buscavam definir a mulher como um ser naturalmente destinado ao exercício da maternidade, e esses discursos se conectavam diretamente com o casamento. Logo, em prol do "bem da nação", do progresso e do "futuro da espécie", os eugenistas e médicos se apresentavam como legisladores sociais preparados pelo conhecimento científico para prescrever as normas adequadas no que diz respeito ao comportamento e à moral (Rohden, 2003).

Percebe-se, logo, que é fundamental pensar a respeito do gênero no contexto de disseminação de políticas eugênicas, sendo aqui o gênero entendido como elemento que, baseado na diferença percebida entre os sexos, constitui as relações sociais (Scott, 1995). A bibliografia tem chamado a atenção para o fato de que os eugenistas deram centralidade ao controle dos corpos femininos e de que muitas das suas ações educativas se voltavam para o público feminino.[1] Entretanto, um aspecto da história da eugenia ainda pouco explorado é a participação das mulheres no movimento eugênico.

Existem estudos sobre a atuação feminina em outros países, principalmente em relação aos EUA e às ações de Margaret Sanger[2] (1879-1966) para o controle da natalidade, mas, no que diz respeito ao Brasil, ainda existe uma lacuna a ser explorada. A hipótese que levanto é que as mulheres não foram apenas alvo de políticas eugênicas, mas também agentes desse movimento, quebrando com a ideia de que as mulheres latino-americanas só se fizeram presentes nos círculos eugênicos na posição de auxiliares e de um mero objeto da eugenia.

No que tange às fontes, venho trabalhando com uma série de teses, livros e conferências de mulheres como Maria Lacerda de Moura (1887-1945), Adalzira Bittencourt (1904-1976), Sylvia Serafim (1902-1936), Lily Lages (1907-2003), Ítala Silva de Oliveira (1897-1984), Juana Lopes (1892/3-?) e Eunice Penna Kehl (1901-1980), além de artigos escritos por mulheres e publicados nos periódicos *Boletim de Eugenia*[3] e *Boletim de Educação Sexual*.[4] Aqui darei destaque à produção de duas mulheres, Ítala Silva de Oliveira e Eunice Penna Kehl, comparando suas atuações.

Eunice Kehl era esposa do eugenista Renato Kehl (1889-1978)[5] e filha do sanitarista Belisário Penna (1868-1939). A julgar pelos seus diários,[6] era uma mulher sempre em busca de novos projetos. Dava aulas de alemão e fundou, junto com uma amiga e sócia, uma academia de ginástica para mulheres. Em seus diários, Eunice relata que tinha vontade de seguir a carreira de escritora, mas que não o fazia pois, segundo ela, "a mulher não pode dedicar-se à literatura, sem prejuízo para as suas obrigações" (Kehl, 2022, p.19). Além disso, ela relata que outra coisa que a afastava da vida literária era a sua crença de que um casal não poderia ser feliz "quando marido e mulher lutam no mesmo campo de competições" (p.19). Segundo o relato de Eunice, o homem não se conforma com o sucesso literário da esposa e, por mais que a ame, jamais ficaria satisfeito em ser "o marido de fulana de tal" (p.19). Assim, ela conclui que, por prezar muito a sua tranquilidade e harmonia conjugal, ela nem mesmo tenta a experiência de ser escritora.

Em alguns momentos de seus diários, ela relata que ajudava Renato Kehl a corrigir capítulos de seus livros e que o marido queria que ela fundasse uma "associação pró-cultura feminina" (Kehl, 2022, p.228). Além disso, Eunice (Kehl, 1935) prefaciou um importante livro de Kehl, *Como escolher um bom marido*. Trata-se de um manual sobre como escolher um cônjuge de acordo com os princípios eugênicos, publicado em 1923 e reeditado em 1935, com base em conferências realizadas pelo próprio Renato Kehl.

Ao longo do prefácio, é possível perceber que Eunice adota um tom de aconselhamento, pois ela trata as leitoras em potencial como amigas que, por meio dos seus conselhos, seriam iniciadas no conhecimento eugênico. O texto se inicia com a autora expondo que o livro havia sido escrito por um "ilustre médico patrício" (Kehl, 1935, p.7), ou seja, um homem de atitudes nobres e distintas, merecendo, assim, ter os argumentos trazidos naquele livro respeitados. De acordo com Eunice, as leitoras não demorariam a perceber que o tema tratado na publicação era de grande importância e que a eugenia era uma ciência "digna do interesse de todas as moças inteligentes e cultas" (p.8).

Segundo a autora, sendo ela uma mulher que se dedicava há muito tempo às questões referentes ao progresso feminino e, sobretudo, às questões que diziam respeito ao papel da mulher mãe, dona de casa, educadora e propagandista dos "valiosos e alevantados princípios ditados pela eugenia" (Kehl, 1935, p.8), reservava todos os dias um tempo dentre seus estudos para ler sobre o que ela chama de "cruzada pela melhoria progressiva, quer física, quer intelectual e moral dos nossos semelhantes" (p.8).

Nos seus diários, ela escreve sobre a importância da educação para as mulheres e, frequentemente, aponta os livros que estava lendo. Em um texto de 31 de março de 1935, escreve:

> Minha memória é fraca por falta de educação, pois minha instrução foi das mais rudimentares. Depois dos trinta anos, quando se deu minha emancipação intelectual, é que comecei a ler e ter contato com os grandes espíritos. Vivo, até hoje, num deslumbramento, descobrindo cada dia um pedaço deste mundo, para mim inteiramente novo: o mundo do pensamento humano (Kehl, 2022, p.26).

No prefácio do livro *Como escolher um bom marido*, Eunice relata que a leitura sobre eugenia a agradava tanto, que ela chegava até a se esquecer dos livros de romance. Na opinião de Eunice, os livros de romance eram quase todos escritos com o objetivo de fazer "pseudoestudos psicológicos" (Kehl, 1935, p.8) ou de tratar de assuntos referentes aos problemas do adultério, o que para ela era algo ignorável.

Eunice também relata que foram os livros *Eugenia e medicina social* (1920) e *Melhoremos e prolonguemos a vida* (1923), ambos escritos por Renato Kehl, os responsáveis por despertar nela o gosto pelo estudo da eugenia. Segundo ela, se as leitoras também estudassem sobre a ciência eugênica, logo perceberiam que os males que afligem a humanidade e que semeiam dores e lutos “tiveram como causa primordial a doença, a tara ou o vício transmitido por indivíduos que se casaram em más condições de saúde” (Kehl, 1935, p.9).

A autora também defende o exame médico pré-nupcial obrigatório[7] como medida profilática. Nas palavras de Eunice, uma vez que lhe foi revelado que as doenças e os casamentos realizados em más condições de saúde eram, em grande parte, a causa das mazelas humanas, ela tomou a deliberação de propagar os princípios eugênicos relativos ao casamento. Em sua opinião, não há dúvidas quanto às vantagens do exame pré-nupcial para garantir o melhor estado de saúde das gerações futuras.

Segundo Eunice, as leitoras, por não passarem o dia “a ler romances, a polir as unhas, a preocupar-se com o mundanismo fútil”, por levarem uma “vida venturosa de moça sábia, bela e feliz” e serem dotadas de “uma inteligência norteada por um coração amorável e bom” (Kehl, 1935, p.10), reconheceriam o motivo de ser necessário dar atenção à questão da escolha do marido tratada no livro e a cooperar para a propagação da ciência eugênica entre as mulheres.

Com atuação diferente de Eunice Kehl, temos a médica sergipana Ítala Silva de Oliveira. Bacharel em *sciencias* e letras, professora e obstetriz, Ítala de Oliveira defendeu sua tese na Faculdade de Medicina da Bahia em 1927, intitulada *Da sexualidade e da educação sexual*. Conforme é anunciado em uma edição de abril de 1929 do *Boletim de Eugenia*, a médica representou a Faculdade de Medicina da Bahia no primeiro Congresso Brasileiro de Eugenia,[8] um evento científico importante não somente para o movimento eugênico como também para a própria história intelectual brasileira.

Ítala de Oliveira (1927) defende a tese de que ignorância e pureza jamais foram sinônimas. Seu argumento é que as conquistas crescentes do feminismo e o contato contínuo dos dois sexos nas fábricas, por exemplo, “estão a pedir nos programas de ensino, ao lado da educação física, da moral e da intelectual, um lugar para a educação sexual” (p.15), para que se possa criar na mulher o sentimento da dignidade própria e no homem o respeito a sua companheira de lutas e trabalhos.

Para a médica, o assunto da educação sexual merece ser tratado, pois ele joga com os destinos da humanidade. Ela defende que essa educação seja estendida às mulheres, pois a mulher sofre mais que o homem nessas questões de amor e sexualidade, “a ela tudo sendo proibido, tudo sendo vedado” (Oliveira, 1927, p.17). Assim, em sua opinião, a mulher também tem direito ao conhecimento, em seu benefício próprio e em benefício dos seus filhos amanhã. Segundo ela, a instrução feminina só traria benefícios à espécie.

Ítala de Oliveira (1927) expõe que seu interesse pelo estudo e aplicação da educação sexual advinha do fato de ela ser também professora, podendo assim transmitir ensinamentos que auxiliassem na constituição de uma boa raça. Segundo ela, muitas queixas seriam evitadas se a educação sexual tivesse a atenção que merece, se ela não fosse “em pleno século das mais engenhosas invenções, um espantalho e uma vergonha” (p.15).

Na tese, Ítala de Oliveira também critica o que ela chama de “ocultamento das funções vergonhosas”, ou seja, das funções sexuais. Para ela, o fato de não se tocar nesses assuntos nos livros de história natural se trata de uma ocultação seletiva que se baseia em premissas morais que impedem as mulheres de falar abertamente sobre sexualidade. Na opinião da médica, o conhecimento das funções orgânicas dos sexos não tiraria a pureza de ninguém. Para ela, era importante expor para as jovens que o desejo sexual era algo natural, mas também era importante a educação sexual, pois por meio da educação seria possível blindar a população contra a degeneração.

O feminismo também era tema discutido, e, de acordo com Ítala, vinha sendo mal interpretado, mesmo pelas próprias mulheres. Segundo ela, o feminismo não queria transformar as mulheres em homens, ele apenas queria dar a elas seus direitos humanos, "tornando sua posição independente, com direitos, deveres e responsabilidades, correspondentes às atribuições normais na sociedade" (Oliveira, 1927, p.166)

## Considerações finais

Podemos concluir que o discurso médico eugênico afirmava que os atributos femininos, por uma disposição natural, eram voltados para a maternidade e para o espaço privado. Os eugenistas argumentavam em favor de uma suposta relevância do papel materno para a "evolução" e elevação da raça. Entretanto, as mulheres não foram apenas objetos da eugenia, mas também agentes desse movimento, atuando de diferentes formas e discutindo questões como maternidade, casamento e educação sexual, conforme foi mostrado pelos exemplos de Eunice Kehl e Ítala de Oliveira.

A eugenia tem despertado o interesse de vários historiadores brasileiros, visto que se mostra como um objeto de estudo privilegiado. Embora já exista vasta historiografia dedicada ao tema, o assunto não está, contudo, esgotado, uma vez que há falta de estudos aprofundados sobre a atuação feminina nesse movimento. Com isso, abre-se espaço para a realização de estudos com novos enfoques, perspectivas e abordagens. Logo, acredito que este estudo pode agregar para a ampliação da temática do movimento eugênico.

### NOTAS

[1] Destaco aqui as obras: *A hora da eugenia: raça, gênero e nação na América Latina* , de Nancy Stepan (2005); *Progressive mothers, better babies: race, public health, and the state in Brazil, 1850-1945*, de Okezi Otovo (2016); *"Ser mãe é uma ciência": mulheres, médicos e a construção da maternidade científica na década de 1920*, de Maria Martha de Luna Freire (2008).

[2] Margaret Sanger ficou conhecida por apoiar movimentos favoráveis ao controle da natalidade e por lutar pela criação da primeira pílula anticoncepcional. Sanger foi uma personagem importante em prol de medidas de esterilização eugênica, fundando a American Birth Control League, em 1921. A organização promoveu a fundação de clínicas de controle de natalidade e encorajou as mulheres a controlar sua própria fertilidade (Davis, 2016).

[3] O *Boletim de Eugenia* foi fundado por Renato Kehl e circulou entre 1929 e 1933. No periódico, a participação feminina é bem pequena, mas nele há artigos que tratam da importância da educação sexual e da maternidade consciente.

[4] O *Boletim de Educação Sexual* foi fundado por José de Albuquerque e circulou entre 1933 e 1939. Nele, a participação feminina é mais significativa, sendo encontrados 17 artigos assinados por diferentes mulheres, médicas, escritoras e professoras.

[5] Renato Kehl foi um dos maiores divulgadores da eugenia brasileira, recebendo o título de "pai da eugenia no Brasil".

[6] Os diários de Eunice Kehl correspondentes a 1935 e 1936 foram publicados pela editora Chão em 2022. Os diários se mostram relevantes como fonte histórica, pois, por meio deles, é possível compreender práticas culturais de uma época. Eles permitem ao historiador rastrear maneiras de viver e pensar de determinada época (Cunha, 2015).

[7] O exame médico pré-nupcial proposto pelos eugenistas tinha como objetivo principal impedir uniões entre indivíduos considerados degenerados. O exame era considerado, pela maioria dos eugenistas, um mecanismo importante para se alcançar o melhoramento racial, o que, na visão desses homens, fazia com que a sua obrigatoriedade fosse altamente necessária. Em resumo, era defendido que todos aqueles que tivessem a pretensão de se casar deveriam passar por um exame médico no qual seria avaliada a saúde dos nubentes. Seria então emitido um atestado de comprovação de sanidades física e mental que deveria ser apresentado como um dos documentos necessários para a realização do casamento civil (Vieira, 2022).

[8] O Congresso Brasileiro de Eugenia aconteceu entre 1 e 6 de julho de 1929, em comemoração ao centenário da fundação da Academia Nacional de Medicina. Sob a presidência de Roquette-Pinto, o congresso foi realizado na sede da Faculdade de Medicina do Rio de Janeiro.
